# Glacier Algae: A Dark Past and a Darker Future

**DOI:** 10.3389/fmicb.2019.00524

**Published:** 2019-04-04

**Authors:** Christopher J. Williamson, Karen A. Cameron, Joseph M. Cook, Jakub D. Zarsky, Marek Stibal, Arwyn Edwards

**Affiliations:** ^1^ Bristol Glaciology Centre, School of Geographical Sciences, University of Bristol, Bristol, United Kingdom; ^2^ Institute of Biological, Environmental & Rural Sciences, Aberystwyth University, Aberystwyth, United Kingdom; ^3^ Department of Geography, The University of Sheffield, Sheffield, United Kingdom; ^4^ Department of Ecology, Faculty of Science, Charles University, Prague, Czechia

**Keywords:** glacier algae, Streptophytes, albedo, terrestrialization, ice

## Abstract

“Glacier algae” grow on melting glacier and ice sheet surfaces across the cryosphere, causing the ice to absorb more solar energy and consequently melt faster, while also turning over carbon and nutrients. This makes glacier algal assemblages, which are typically dominated by just three main species, a potentially important yet under-researched component of the global biosphere, carbon, and water cycles. This review synthesizes current knowledge on glacier algae phylogenetics, physiology, and ecology. We discuss their significance for the evolution of early land plants and highlight their impacts on the physical and chemical supraglacial environment including their role as drivers of positive feedbacks to climate warming, thereby demonstrating their influence on Earth’s past and future. Four complementary research priorities are identified, which will facilitate broad advances in glacier algae research, including establishment of reliable culture collections, sequencing of glacier algae genomes, development of diagnostic biosignatures for remote sensing, and improved predictive modeling of glacier algae biological-albedo effects.

## Introduction

Glacier surfaces are home to diverse and active microbial communities (Hodson et al., 2008; Stibal et al., 2012; Anesio et al., 2017). The coincidence of liquid water and sunlight during summer months supports substantial phototrophy at the glacial surface. One key group of supraglacial primary producers are heavily pigmented green microalgae of the Mesotaeniaceae (Zygnematophyceae, Streptophyta), first documented by Adolf Erik Nordenskiöld during his explorations of Greenland (Nordenskiöld, 1872). The importance of these microalgae is manifested both through the insights they can provide into the development of the world’s terrestrial flora and their acceleration of glacier wastage, expanding the rationale for their study. This minireview aims to synthesize current knowledge on these algae, drawing on recent research pertaining to their phylogeny, physiology, ecology, and impacts in supraglacial systems. To avoid confusion with the better known “ice algae” associated with sea ice habitats (Boetius et al., 2015) or chlorophyte algae associated with snow pack environments (Hoham and Duval, 2001), we propose here the adoption of “glacier algae” to refer to this group of surface ice inhabiting Streptophytes.

## Taxonomy and Phylogenetics

**Figure 1:**
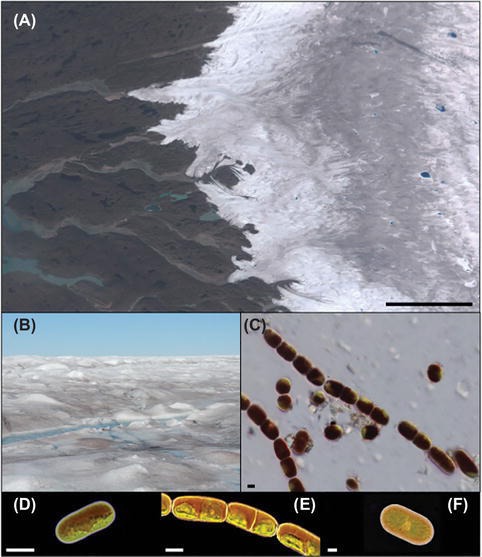
Glacier algae and the supraglacial environment: **(A)** RGB composite image of the southwestern Greenland Ice Sheet (GrIS) margin near Kangerlussuaq, derived from European Space Agency Sentinel-2 data. Note the conspicuous “dark zone” running parallel to the ice sheet margin for which glacier algal blooms are thought responsible. **(B)** GrIS surface ice within the dark zone dominated by a glacier algal bloom during the 2016 ablation season. **(C)** Glacier algae assemblage sampled from the surface of the GrIS. **(D)** *M. berggrenii*; **(E)** *A. nordenskiöldii*; and **(F)** *C. brebissonii* (contributed by Nozomu Takeuchi). Scale bars are 20 km **(A)** and 10 μm **(C–F)**.

For almost 150 years, numerous reports have described algal communities that reside on bare ice surfaces around the globe (e.g., Nordenskiöld, 1872; Kol and Taylor, 1942; Yoshimura et al., 1997; Remias et al., 2009; Yallop et al., 2012). The low diversity of assemblages has resonated throughout more than 20 studies, with just three key species typically present (Figure 1); the chained filamentous *Ancylonema nordenskiöldii*, two varieties of the unicellular *Mesotaenium berggrenii* distinguishable by their size and number of chloroplasts within freshly divided cells (Kol, 1968; Ling and Seppelt, 1993; Remias et al., 2009) and the unicellular Cylindrocystis brebissonii. Descriptions of these cylindrical cells, which contain chloroplasts, pyrenoids, and “dark violet cell sap,” have been provided by Kol and Taylor (1942) and Yoshimura et al. (1997) based on their explorations in Alaska and the Himalayas, respectively, with more recent ecophysiological and ultrastructural descriptions provided by Remias et al. (2009, 2012a,b). While other algal species have also been reported from glacial ice surfaces, e.g., Chlamydomonas nivalis and *Raphidonema sempervirens*, these are assumed remnants of, or depositions from, other environmental niches such as snow or soils (Takeuchi, 2013; Lutz et al., 2017) and are not true ice environment specialists.

**Figure 2:**
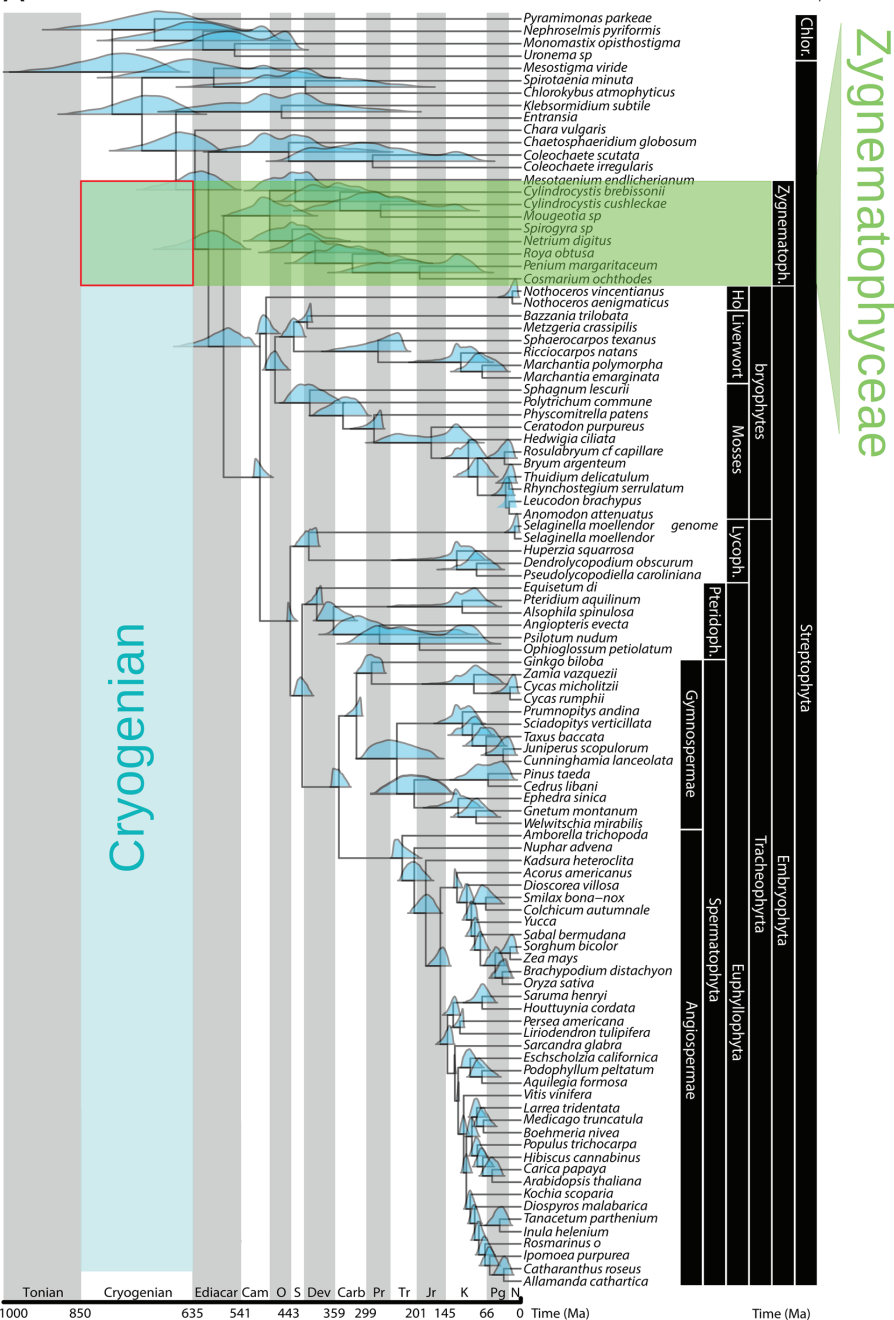
Streptophyte phylogeny and congruent age estimates, highlighting the Zygnematophyceae as sister lineage to land plants, and the timing of the Chlorophyte/Streptophyte division during the Cryogenian period (adapted with permission from Morris et al., 2018). Blue density plots show 95% highest posterior density of age estimates.

Glacier algae belong to the Streptophyta, a subphylum of the Chloroplastida that split from the Chlorophytes during the Cryogenian geologic period, when the Earth was extensively covered with snow and ice (Lewis and McCourt, 2004; Becker and Marin, 2009; Leliaert et al., 2012; Becker, 2013) (Figure 2). Streptophytes are composed of the Charophytes, a paraphyletic assemblage of freshwater algae in which glacier algae reside, and all land plants (Leliaert et al., 2012). Of the former, the majority of diversity is contained within the Zygnematophyceae, which includes coccoid, filamentous, and colonial forms distinguished by the absence at any stage in the life cycle of flagella and their unique method of sexual reproduction, conjugation of non-flagellated gametes (Gontcharov, 2008; Guiry, 2013). Members are found in both aquatic (solely freshwater) and terrestrial habitats (Lewis and McCourt, 2004) and can occupy a number of extreme environments including acid bogs, alkaline streams, desert crusts, snow, and ice (Hall et al., 2008). All three glacier algae species belong to the Zygnematophyceae, within the family Mesotaeniaceae (the saccoderm desmids) (Gontcharov, 2008; Hall et al., 2008; Remias et al., 2009, 2012a).

The phylogenetic positioning of the Zygnematophyceae as the closest living relatives to extant land plants (Wodniok et al., 2011), and partial support for the Mesotaeniales as basal among the Zygnematophyceae (Wickett et al., 2014; De Vries et al., 2016), leads to the inference that glacier algae are informative for early land plant evolution. The process of land plant evolution from within a single freshwater Streptophyte algal lineage represented a singularity in Earth’s history (Wickett et al., 2014; De Vries et al., 2016; De Vries and Archibald, 2018), leading to one of the most profound geobiological transitions in the history of the planet (Dahl et al., 2010; Kump, 2014; Delwiche and Cooper, 2015; Selosse et al., 2015). Exaptations of ancestral Streptophytes to environmental stressors likely favored their transition to land, i.e., adaptations evolved for some purpose in water that later proved advantageous on land (Waters, 2003; Delwiche and Cooper, 2015; De Vries and Archibald, 2018). Adaptations key for the transition from aquatic to terrestrial habitats included the ability to tolerate extremes in temperature, desiccation, irradiance, and UV radiation (Waters, 2003; Delwiche and Cooper, 2015; De Vries and Archibald, 2018), and it is thus conceivable that supraglacial environments may have played a role in driving the evolution of key land plant biological features. If the common ancestor of extant Streptophyte algae and land plants inhabited glacier surfaces, this may represent a fundamental change in the way we think about the driving forces behind the processes of land plant terrestrialization.

## Ecology and Physiology

The upper surface (~2 cm) of supraglacial ice in which glacier algal blooms occur is characterized by extremes in environmental stressors. During summer ablation seasons, positive air temperatures and significant short-wave radiation drive ice surface melting, producing a hydrologically dynamic environment (Muller and Keeler, 1969; Cook et al., 2016). Concomitantly, photoinhibitory levels of photosynthetically active radiation (PAR, 400–700 nm, e.g., ~1,700 μmol photons m^−2^ s^−1^ on a sunny, cloudless day; Yallop et al., 2012) and UV stress (Morgan-Kiss et al., 2006) couple with low nutrient concentrations (e.g., <1 μM P l^−1^ Hawkings et al., 2016; 1.3 μM DIN l^−1^ Wadham et al., 2016) and diurnal freeze–thaw cycles (Cook et al., 2016) to produce an extremely challenging environment. During winter periods, surface ice communities further experience sub-zero temperatures, complete darkness, and burial under snow packs.

Blooms of algae on glacier and ice sheet surfaces have now been reported from across the cryosphere, including Antarctica (Ling and Seppelt, 1993), Alaska (Takeuchi, 2001, 2013; Ganey et al., 2017), Siberia (Takeuchi et al., 2006, 2015; Tanaka et al., 2016), the Himalayas (Yoshimura et al., 1997), Svalbard (Remias et al., 2012a), and Greenland (Uetake et al., 2010; Yallop et al., 2012; Stibal et al., 2017; Williamson et al., 2018), indicating their apparent ubiquity in supraglacial systems. Blooms initiate following snow line retreat, with algal biomass observed to increase in surface ice through time (Stibal et al., 2017; Williamson et al., 2018). In contrast to snow algae (Hoham and Duval, 2001), the absence of a flagellated life stage prevents active motility of glacier algae, and thus, colonization of new ice environments during bloom events is likely dependent on local hydrological or aeolian forcing (Kristiansen, 1996). On the Greenland Ice Sheet (GrIS), population doubling times have been estimated at 3.75–5.5 days (Stibal et al., 2017; Williamson et al., 2018), with cell densities observed to range from 9.1 × 10^4^ to 29.5 × 10^4^ cells ml^−1^ at marginal locations (Yallop et al., 2012), from <100 to 8.5 × 10^4^ cells ml^−1^ ~30 km into the southwesterly region of the ice sheet (Stibal et al., 2017), and from 1.6 × 10^4^ cells ml^−1^ to 0 cells ml^−1^ from ~30 km inland to the snow line (Williamson et al., 2018). The influences on spatial patterning in biomass are multifaceted. Observations of algal biomass on mountain glaciers (e.g., Yoshimura et al., 1997; Takeuchi and Kohshima, 2004; Takeuchi et al., 2009) show declines in biomass with increasing altitude, while observations from the GrIS’s “dark zone” (a conspicuous area of dark ice that appears across the west and southwestern sectors of the ice sheet each summer; Figure 1; Wientjes and Oerlemans, 2010) show a decrease in biomass away from the ice sheet margin (Williamson et al., 2018). Considered jointly, these intimate that longer melt seasons support algal biomass development through promoting solar radiation input, nutrient availability, and diminished snow cover (Yoshimura et al., 1997). Decreases in biomass can be driven by rainfall-associated flushing events (Stibal et al., 2017), and biomass is potentially restricted close to the terminus of glaciers by mineral dust covering that can limit photosynthesis and/or by increased meltwater flushing on steeper slopes (Takeuchi, 2013). Interspecific interactions also influence the relative dominance of glacier algae at the glacier scale, with specialists dominating more stable ice environments and generalist species becoming dominant in areas characterized by less stable conditions, e.g., frequent changing between snow and ice environments (Yoshimura et al., 1997).

The most visually striking adaptation of glacier algae to their environment is the production of a specialist pigment absorbing ultraviolet and visible light (purpurogallin carboxylic acid-6-*O*-Beta-D-glucopyranoside), contained within lipid bodies and vacuoles occupying a large proportion of the cell (Figure 1; Remias et al., 2009, 2012a,b). In addition to the suite of light-harvesting and photoprotective pigments typical of green microalgae (Remias et al., 2009, 2012b; Williamson et al., 2018), this phenolic pigment is primarily assumed to serve a photoprotective role, shading the underlying chloroplasts from the significant PAR and UV regime apparent in supraglacial systems (Remias et al., 2009, 2012b; Williamson et al., 2018). It also likely serves to convert the abundant light energy to heat, allowing melt water generation local to the cell (Dial et al., 2018). To date, the capacity of glacier algal phenols to provide photoprotection has been indirectly evidenced by a lack of saturation during photosynthesis-irradiance curves (Remias et al., 2012a,b) and fluorescence-based rapid light curves (Yallop et al., 2012), ranging up to 2000 μmol photons m^2^ s^−1^. Given that the photosynthetic machinery is adversely affected by several cold associated stressors (i.e., freezing and desiccation reduce cell membrane fluidity impacting electron transport; low temperatures mimic high-light stress by decreasing the efficiency of metabolic electron sinks; Lyon and Mock, 2014), it is likely that glacier algae purpurogallins serve to protect the cell against multiple stressors.

Little further information on glacier algae adaptations to life in surface ice is available, with this knowledge gap strongly exacerbated by their reluctance to be cultured under laboratory conditions (Remias et al., 2009, 2012a). Though conjugation in *A. nordenskiöldii* field populations has been observed in Svalbard (Remias et al., 2012a,b) and the GrIS (C. Williamson, personal observation), the production of a dormant zygospore does not appear to be an overwintering strategy, with glacier algae observed to overwinter in a non-cyst-like, vegetative state (Remias et al., 2009). This likely permits rapid resumption of physiological activity on initiation of the relatively short summer growth season. Glacier algae also demonstrate increased concentrations of sugars and polyols (i.e., compatible solutes) (Roser et al., 1992; Chapman et al., 1994), consistent with known cold tolerance mechanisms in other psychrophilic microalgae (Welsh, 2006; Casanueva et al., 2010; Lyon and Mock, 2014). However, knowledge on other features typically associated with cold tolerance in microalgae, e.g., membrane fluidity, production of specialist enzymes, “cold-shock” proteins or extracellular polymeric substances, is currently lacking.

## Impacts to Environment

The pigmentation of glacier algae (Remias et al., 2009, 2012b; Williamson et al., 2018) coupled with abundances achieved during summer blooms (Yallop et al., 2012; Stibal et al., 2017; Williamson et al., 2018) can have profound implications for both the physical (melt) and chemical (nutrient and carbon cycling) surface ice environment. Given that albedo, i.e., the probability that light entering ice will be scattered back into the atmosphere rather than absorbed, is an important control of surface ice melting (Box et al., 2012; Tedesco et al., 2016), the processes that serve to darken surface ice (reduce albedo) hold significant potential to impact melting. Through the production of highly absorbing phenolic pigments (see previous section), glacier algae act as effective light absorbing particles (LAPs) and hold large potential to accelerate the melting of glaciers and ice sheets through biologically driven albedo reduction (Takeuchi, 2013; Takeuchi et al., 2015; Cook et al., 2017; Stibal et al., 2017; Tedstone et al., 2017; Ryan et al., 2018). It has been suggested that wider melt zones under warmer climates may provide larger areas for glacier algae colonization, raising the possibility of a melt-enhancing positive feedback (Yallop et al., 2012).

While the impacts of glacier algae-driven albedo reduction remain to be quantified at the scale of glaciers, ice sheets, or across the cryosphere, glacier algal blooms have been suggested by both observational (Yallop et al., 2012; Stibal et al., 2017; Ryan et al., 2018) and modeling efforts (Tedstone et al., 2017) to be responsible for long-term declines in GrIS surface albedo that have paralleled accelerating surface melt, particularly along the western margin of the ice sheet in the “dark zone.” Given that melt of the GrIS is the single largest contributor to global sea level rise (Box and Sharp, 2017; Bamber et al., 2018), the potential contribution of glacial algal blooms to global sea level rise remains a highly active field of research.

Additional to feedbacks on surface melt, glacier algal blooms may also impact carbon and nutrient cycling within surface ice environments, with consequences for downstream ecosystems (Stibal et al., 2012). Glacier algae photosynthesize at surprisingly high rates considering their thermodynamically unfavorable cold environment (Remias et al., 2009, 2012a; Cook et al., 2012; Yallop et al., 2012; Williamson et al., 2018). Recent estimates of glacier algal net productivity in southwestern Greenland ranged from ~0.5 to 1 mg C l^−1^ d^−1^, based on ice containing dense algal communities (~10^4^ cells ml^−1^; Yallop et al., 2012; Williamson et al., 2018). While few attempts have been made to constrain the importance of glacier algae for supraglacial carbon budgets, recent modeling efforts for regions of the southwestern GrIS have highlighted the major contribution that blooms can make to supraglacial carbon fixation (Cook et al., 2012; Williamson et al., 2018), with an average net carbon fixation of ~16 ± 8 kg C km^2^ estimated for the 2016 ablation season (Williamson et al., 2018). This can lead to accumulation of autochthonous organic carbon within glacier algal-rich habitats (Musilova et al., 2017). Labile organic carbon not consumed *in situ* by secondary production may be exported *via* meltwater flushing for utilization within downstream subglacial and periglacial ecosystems (Musilova et al., 2017; Smith et al., 2017).

## Future Research Priorities

Here, we identify four complementary research priorities that will facilitate broad advances in glacier algae research, serving to generate knowledge on the life histories, physiology, ecology, and genomics of the algae themselves, allowing projection of their occurrence and impacts across the cryosphere into the future.

### Establishment of Reliable Culture Collections

Efforts to establish reliable culture collections of glacier algae remain a priority as these will undoubtedly facilitate widespread knowledge advances. To date, short-term incubation studies using freshly collected field material have yielded initial insights into glacier algae physiology (Remias et al., 2012a,b) and are starting to be complemented by *in situ* manipulative experiments; though the latter typically require significant logistical (and thus financial) commitments and are themselves hindered by a lack of equipment optimized for constraining physiology within icy environments. To the best of the collective authors’ knowledge, no group has yet been able to establish a reliable culture collection of the true ice environment specialists *M. berggrenii* and *A. nordenskiöldii*, preventing even the most basic of progress in understanding the life histories of these species or their abilities to thrive under supraglacial conditions. Overcoming this impasse will open up the field to a plethora of new discoveries relating to topics such as physiological tolerances, cold adaptation mechanisms, and the production of novel compounds. This research will also underpin efforts to project the occurrence of glacier algal blooms across the cryosphere and their impacts to surface ice environments and downstream ecosystems. Uniting current efforts and the collective knowledge of disparate research groups may serve to advance this area and minimize duplication of efforts.

### Sequencing of Glacier Algae Genomes

The near absence of genome level data for glacier algae has significantly exacerbated current knowledge gaps for these species. This information void has prevented comparative genomics approaches required to investigate, for example, the evolution of cold adaptation mechanisms across lineages, and has precluded exploration of the importance of supraglacial habitats for the evolution of a terrestrial flora. Given current culturing impasses, metagenomic or single-celled sequencing approaches may be best employed to achieve this goal. Additional to the wealth of information that genome interrogation will provide, the availability of glacier algae reference genomes will facilitate a suite of transcriptomic studies that will significantly advance our understanding of how life functions within icy environments.

### Develop Diagnostic Biosignatures for Glacier Algae Remote Sensing

While an increased understanding of the physiological tolerances of glacier algae derived from culturing studies will improve our ability to model the occurrence and magnitude of glacier algal blooms, the remoteness and vastness of the cryosphere necessitate a remote-sensing approach to validate models and constrain bloom occurrence cryosphere-wide. This will serve to monitor spatial and temporal dynamics in bloom development across a myriad of environmental conditions, providing the data needed to project bloom occurrence into the future. It is also critical for constraining the importance of glacier algae for the mass balance of glaciers and ice sheets and, by extension, their contribution to global sea level rise. For snow algae, Takeuchi et al. (2006) remotely quantified algal biomass using a simple band ratio technique, while Painter et al. (2001) used the chlorophyll absorption feature at 680 nm. Huovinen et al. (2018) demonstrated the potential for spectral mixing analysis to separate biological and mineral components and quantify algal biomass, which outperforms band ratios and specific pigment spectral features. However, this has not yet successfully been applied to glacier algae due to specific challenges related to the optics of the underlying ice and glacier algae pigmentation. The purpurogallin pigment characteristic of glacier algae may well obscure diagnostic spectral features associated with other individual pigments. Secondary spectral features may also need to be identified that distinguish the biosignatures of glacier algae and snow algae, which may co-occur following snow line retreat. There may also be complex optical effects related to the mixing of biological and non-biological LAPs and the highly variable optical properties of the weathered ice surface itself. To date, bio-optics of cryospheric algae have relied upon theoretical estimates of absorption and scattering derived from mixing models that combine *in vivo* absorption coefficients of known pigments (Cook et al., 2017). Empirical measurements of the bulk optical properties of glacier algal cells will be required to validate existing models and constrain realistic biological optical effects, enabling biosignature determination. Future studies should build upon the past successes in the remote sensing of snow algae (Painter et al., 2001; Takeuchi et al., 2006; Huovinen et al., 2018) and also employ multispectral sensors and pair remotely sensed spectral reflectance measurements with sampling of surface ice allowing integrated ground truthing of remote observations.

### Improve Predictive Modeling of Glacier Algae Biological-Albedo Effects

Biological albedo is a potentially significant component of the energy balance of glaciers and ice sheets that is yet to be fully quantified. While several studies have now indicated a primary role for glacier algae in controlling ice albedo in Greenland’s “dark zone” (Yallop et al., 2012; Stibal et al., 2017), quantifying the albedo reduction that can be attributed to glacier algae remains challenging. Difficulties in separating biotic, biogenic, and abiotic albedo reduction in empirical measurements necessitate a theoretical predictive modeling approach (Cook et al., 2017), which itself requires validation through empirical measurements of the bulk single scattering optical properties of glacier algae cells. It will also be important to constrain the vertical distribution of glacier algae within surface ice at the scale of millimeters, given the dramatic effect this can have on albedo reduction in radiative transfer models (see Cook et al., 2017). Currently, the practical limit of ~2 cm vertical sampling resolution for field measurements limits the two-way transfer of information between radiative transfer models and empirical measurements. Improving the integration of measurements with theoretical foundations is a priority to enhance the utility of such models (Cook et al., 2017). This will enable incorporation of the impacts of glacier algal blooms into regional climate models (Benning et al., 2014; Stibal et al., 2017; Noël et al., 2018), allowing improved estimation of their contribution to global sea level rise under future climate scenarios.

## Author Contributions

CW coordinated this mini-review, with all authors contributing sections and editing the entire manuscript. AE was responsible for the original concept.

### Conflict of Interest Statement

The authors declare that the research was conducted in the absence of any commercial or financial relationships that could be construed as a potential conflict of interest.
